# Water-soluble pristine C_60_ fullerenes attenuate isometric muscle force reduction in a rat acute inflammatory pain model

**DOI:** 10.1186/s12891-023-06719-w

**Published:** 2023-07-25

**Authors:** Danylo O. Zavodovskiy, Nataliya V. Bulgakova, Inna Sokolowska, Yuriy I. Prylutskyy, Uwe Ritter, Olga O. Gonchar, Alexander I. Kostyukov, Oleh V. Vlasenko, Kamila Butowska, Agnieszka Borowik, Jacek Piosik, Andriy Maznychenko

**Affiliations:** 1 Bogomoletz Institute of Physiology, Bogomoletz Str. 4, Kyiv, 01024 Ukraine; 2 Gdansk University of Physical Education and Sport, Kazimierza Gorskiego Str. 1, Gdansk 80- 336, Gdansk, Poland; 3 ESC “Institute of Biology and Medicine”, Taras Shevchenko National University of Kyiv, Volodymyrska Str. 64, Kyiv, 01601 Ukraine; 4 Institute of Chemistry and Biotechnology, Technical University of Ilmenau, Weimarer Str. 25, 98693 Ilmenau, Germany; 5 Laboratory of Experimental Neurophysiology, National Pirogov Memorial Medical University, Vinnytsya, Ukraine; 6 Laboratory of Biophysics, Intercollegiate Faculty of Biotechnology UG-MUG, Abrahama 58, Gdansk, 80-307 Poland

**Keywords:** C_60_ fullerene nanoparticles, Muscle contraction, Inflammation, Rat

## Abstract

**Background:**

Being a scavenger of free radicals, C_60_ fullerenes can influence on the physiological processes in skeletal muscles, however, the effect of such carbon nanoparticles on muscle contractility under acute muscle inflammation remains unclear. Thus, the aim of the study was to reveal the effect of the C_60_ fullerene aqueous solution (C_60_FAS) on the muscle contractile properties under acute inflammatory pain.

**Methods:**

To induce inflammation a 2.5% formalin solution was injected into the rat triceps surae (TS) muscle. High-frequency electrical stimulation has been used to induce tetanic muscle contraction. A linear motor under servo-control with embedded semi-conductor strain gauge resistors was used to measure the muscle tension.

**Results:**

In response to formalin administration, the strength of TS muscle contractions in untreated animals was recorded at 23% of control values, whereas the muscle tension in the C_60_FAS-treated rats reached 48%. Thus, the treated muscle could generate 2-fold more muscle strength than the muscle in untreated rats.

**Conclusions:**

The attenuation of muscle contraction force reduction caused by preliminary injection of C_60_FAS is presumably associated with a decrease in the concentration of free radicals in the inflamed muscle tissue, which leads to a decrease in the intensity of nociceptive information transmission from the inflamed muscle to the CNS and thereby promotes the improvement of the functional state of the skeletal muscle.

## Background

Attention to C_60_ fullerenes in biomedical investigations, is determined its ability to interact with biomolecules and penetrate through the cell membrane [1–3]. They exhibit powerful antioxidant properties [4–7] and, being non-toxic (at low concentration at least) [3, 8] exert specific health effects. One of these influences is the manifestation of anti-inflammatory effects of C_60_ fullerene nanoparticles. The number of works on this topic is small and mainly concerns toxicological studies, including the effect on respiratory toxicity and immunotoxicity [8–12]. It has also been shown that water-soluble polyhydroxylated C_60_ fullerene nanoparticle inhibits macrophage activation and development of osteoarthritis in rats [13]. It is known, common symptom of inflammatory muscle diseases which leads to a significantly reduced quality of life is a myalgia [14]. In this regard, study of the C_60_ fullerene aqueous solution (C_60_FAS) effect on skeletal muscle inflammation is considerable interest. It was previously shown that C_60_FAS may have a positive effect on the mechano-kinetic characteristics of the soleus muscle during the development of chronic inflammation induced by ischemic or mechanical muscle damage [15, 16]. However, the effect of C_60_FAS on the contractile characteristics of muscles under the development of acute inflammation is still unclear.

It is known that inflammation is a natural biological process that takes places as response to harmful stimuli on tissues. As a rule it is a curative process with direct to wound healing and infections. However, one of the components of inflammation is oxidative stress when reactive oxygen species (ROS) are formed including free radicals, peroxides, oxygen ions and also excess lactic acid, and lipid peroxidation. This process may be deleterious if left unchecked. C_60_ fullerenes can counteract ROS through powerful antioxidant capabilities and this way may inhibit inflammation [16, 17]. In our previous biochemical studies, we observed a significant decrease in ROS formation and concentrations of oxidative stress markers after C_60_FAS administration under conditions of skeletal muscle fatigue development as well as after muscle atrophy or ischemia [18–21].

Formalin-induced acute inflammatory pain model is one of the simplest and most reliable methods. This method is known as the “formalin test” and was first described in 1977. The behavioral response of cats and rats to injection of 5% formalin solution has been used to assess pain and pain relief [22, 23]. This test is still widely used in scientific research. Previously, Lei and You used in their work intramuscular injections of 2.5% formalin into the gastrocnemius muscle in a volume of 25 to 200 µL to induce acute inflammatory pain [24, 25]. Taking into account the possibility of intramuscular administration, and due to the simplicity and reliability of this method, we used formalin injections to induce acute inflammatory pain of the triceps surae (TS) muscle in rats.

Based on our previous works in the field of C_60_FAS application, we hypothesize that water-soluble C_60_ fullerenes would have a significant impact on the increase TS muscle contractile properties under condition of an acute inflammatory pain in rats. Thus, the aim of the study was to revealed effect of the C_60_FAS on isometric contraction of the TS muscle (the muscle that is most involved in daily life) in a rat after formalin administration into this muscle.

## Methods

### Sample preparation and characterization

A highly stable C_60_FAS was prepared and characterized [26] in the Institute of Chemistry and Biotechnology, Technical University of Ilmenau (Germany). For the C_60_FAS preparation at a maximum concentration of 0.15 mg/ml we used a saturated solution of pristine C_60_ fullerene (purity > 99.99%) in toluene with a C_60_ molecule concentration corresponding to maximum solubility near 2.9 mg/ml, and the same amount of distilled water in an open beaker. The two phases formed were treated in ultrasonic bath. The procedure was continued until the toluene had completely evaporated and the water phase became yellow colored. Filtration of the aqueous solution allowed to separate the product from undissolved C_60_ fullerenes [27, 28]. C_60_FAS was stable at 4 ^0^ C for 18 months.

The atomic force microscopy (AFM) was performed to determine the size of C_60_ fullerene particles in aqueous solution. Measurements were done with the “Solver Pro M” system (NT-MDT, Russia). A drop of investigated solution was transferred on the atomic-smooth substrate to deposit layers. Measurements were carried out after complete evaporation of the solvent. For AFM studies, a freshly broken surface of mica (SPI supplies, V-1 grade) was used as a substrate. Measurements were carried out in a semicontact (tapping) mode with AFM probes of the RTPESPA150 (Bruker, 6 N/m, 150 kHz) type [20, 21].

**Fig. 1:**
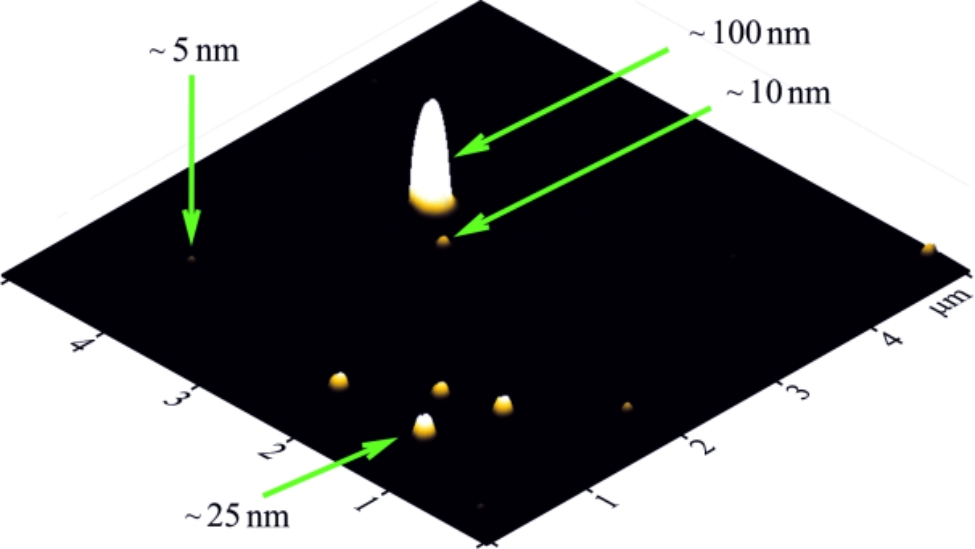
AFM image of C_60_ fullerene nanoparticles on the mica surface (concentration 0.15 mg/ml)

To understand a behavior of C_60_ fullerene in the biological medium (at the levels of cell, tissue and organ) and its interaction with biological targets, it is necessary to know exactly its distribution in size and stability of colloid dispersion at a fixed concentration because the biomedical effects of the C_60_ fullerene particles directly depend on these properties. In this regards, the prepared C_60_FAS was characterized by AFM technique. The study of C_60_ fullerene films deposited from an aqueous solution revealed a high degree of molecules dispersion in solution. It turned out that prepared C_60_FAS contains both single C_60_ fullerene and its labile nanoaggregates with size of ~ 5–100 nm (Fig. 1). Thus, C_60_FAS is a polydisperse colloidal nanosystem. The majority of C_60_ molecules were located chaotically and separately along the surface or in the form of bulk clusters consisting of several tens C_60_ molecules. Such arrangement of C_60_ molecules formed because of electrostatic repulsion between them: the zeta potential value was − 25.3 mV at room temperature, indicating a high solute stabilization [26].

### Procedure and experimental groups

Male Wistar rats weighing 200–250 g were used in the study. The animals were purchased from a state-controlled animal farm through the common animal facility of Bogomoletz Institute of Physiology (Kyiv, Ukraine). The experimental animals were housed in Plexiglas cages and kept in an air-filtered and temperature-controlled (21 ± 1 °C) room under 12-h light/12-h dark conditions. Rats received a standard pellet diet and water *ad libitum*. All procedures complied with the ARRIVE guidelines. The use of the animals was approved by the Biomedical Ethics Committee of the Bogomoletz Institute of Physiology and performed in accordance with the European Union Directive of 22 September 2010 (2010/63/EU) for the protection of animals used for scientific purposes.

**Fig. 2:**
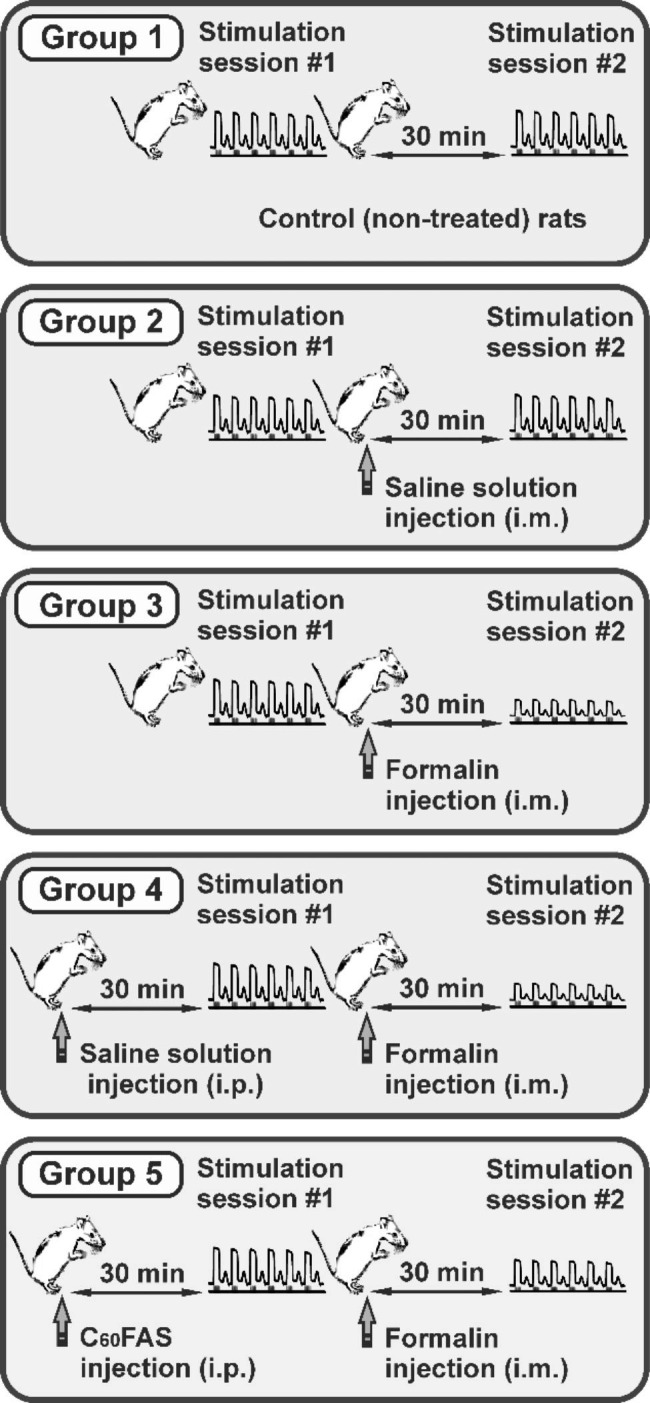
Schematic representation of the study. The variants of saline solution, formalin and C_60_FAS injection, as well as the moments of stimulation are marked in the figure

The animals were randomly divided into 5 groups (Fig. 2). Animal groups: 1 – Control (non-treated) animals (n = 6); 2 – vehicle-treated animals (rats with intramuscular (i.m.) saline solution injection, n = 6); 3 – formalin-treated rats, n = 6; 4 – vehicle-pretreated animals (rats with a preliminary (30 min before formalin application) intraperitoneal (i.p.) injection of 0.2 ml of saline solution, n = 6); 5 – C_60_FAS-pretreated animals (rats with a preliminary (30 min before formalin application) i.p. injection of 0.2 ml (0.15 mg/kg) of C_60_FAS (n = 6) [18].

All animals were subjected two sessions (before and 30 min after formalin injection) of electrical stimulation of *n. tibialis* (tibial nerve); mechanogram of the TS muscle contractions (muscle tension) was recorded during stimulation. The first session of electrical stimulations was carried out without any injection/treatment and sat as a control, whereas second session was used for testing of the muscle tension during inflammation development or it treatment. Inflammation was induced (for 30 min) by injection of 2.5% formalin (“Merck”, Germany) solution into the triceps surae muscle [24, 25]. Injections of the solution were performed under full anesthesia (ketamine/xylazine, see below) into the left TS muscles (two injections of 25 µl into each head).

It is important to note that the doses of the C_60_FAS used in the investigation do not present any acute or sub-acute toxicity in animals. So, accordingly our previous study, the maximum tolerated dose of C_60_FAS was 721 mg/kg for i.p. administration to mice [3].

The animals were anaesthetized (i.p.) with ketamine (100 mg/kg “Pfizer”, USA) combined with xylazine (10 mg/kg, “Interchemie”, Holland). The TS muscle was separated from the surrounding tissue, their tendons were detached at the distal insertions, and a small bone chip from the heel was left behind. The *n. tibialis* was separated from the tissue and cut proximally, and all branches of the nerve, except nerves innervating the TS, were cut. This nerve was mounted on a bipolar platinum wire electrode for electrical stimulation. The hindlimb muscles and nerves were covered with paraffin oil in a pool formed by the skin flaps. The ECG and heart rate were continuously monitored. Pools with mineral oil were maintained at 37–38 °C using radiant heat [18, 19]. The TS muscle was connected via the Achilles tendon to the servo-control muscle puller. Immediately after the experiment, rats were euthanized using high dose of pentobarbital sodium (“Nembutal”, USA).

### Electrophysiology and tensometry

**Fig. 3:**
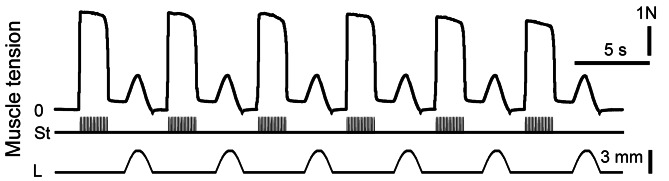
Strength of the triceps surae muscle contractions is performed during first (control) session of electrical stimulations of the *n. tibialis*; the stimulation pattern one of the rat belonging to the non-pretreated group of animals and presented as example. N – muscle force in Newton, st – stimulation mark, L – muscle stretching, mm

To induce muscle contraction, two sessions of 6 series (2 s duration) of intermittent high-frequency electrical stimulations separated by rest intervals of 4 s were used. Each series consisted of trains of 0.2 ms rectangular pulses at a rate of 40 s^− 1^ [29, 30]. The stimulus current was set to 1.3–1.5 times higher than the motor threshold. At the end of the stimulation, the muscle was stretched, and the changes in length had a bell-shaped form (one period of 4 Hz sinusoidal signal with corresponding phase locking) with 3 mm amplitude and 2 s duration (Fig. 3). This stimulation pattern was used to eliminate the effect of fatigue on the inflamed muscle. The muscle reaction to the stretching appeared to be a tension increase after continuous stimulation. These stretches were applied before the post-stimulation twitches to remove, or at least diminish, the after-effects remaining from continuous stimulation [29]. A linear motor under servo-control was used as the muscle puller. The muscle tension was measured by semi-conductor strain gauge resistors glued on a stiff steel beam mounted on the moving part of the linear motor. The stiffness of the puller exceeded 0.06 N/mm, whereas the time constants of the length transients did not exceed 60 ms. The command signal to the muscle puller was derived from a DAC and was adjusted by a scaling amplifier and low-pass filter (0–100 Hz) [18, 19]. In parallel, two analogue signals (muscle tension and length) and pulse signals (stimulation pulses) were sampled via corresponding ADC channels. The signals were collected by PC using an input-output interface device (CED Power 1401) with 12-bit resolution [18, 19].

Data acquisition was performed using the program “Spike2” (CED). Input signals were digitized at rates of 5 kHz (muscle tension) and 1 kHz (other signals). Data analysis, including statistical treatment and graph plotting, was performed using the program Origin 8.5 (Origin Lab Corp., USA) [18, 19].

### Statistical analysis

In the electrophysiological study, each stimulation sessions (6 series) was averaged. The average value of the muscle tension of the first 6 series was set to 100%, and the other series (after inflammation or treatment) were averaged and normalized in relation to ones and presented graphically.

Mean values ± SD (standard deviation) of the TS muscle strength before and after muscle inflammation or after muscle inflammation with preliminary C_60_FAS treatment were compared using one-way analysis of variance (ANOVA). A Bonferroni *post hoc* analysis was used to determine the intergroup differences. The level of significance was set at *p* < 0.05. Shapiro-Wilk test was used to test the normality of the data distribution, and the homogeneity of variance was assessed using Levene’s test for Equality of Variances.

## Results

**Fig. 4:**
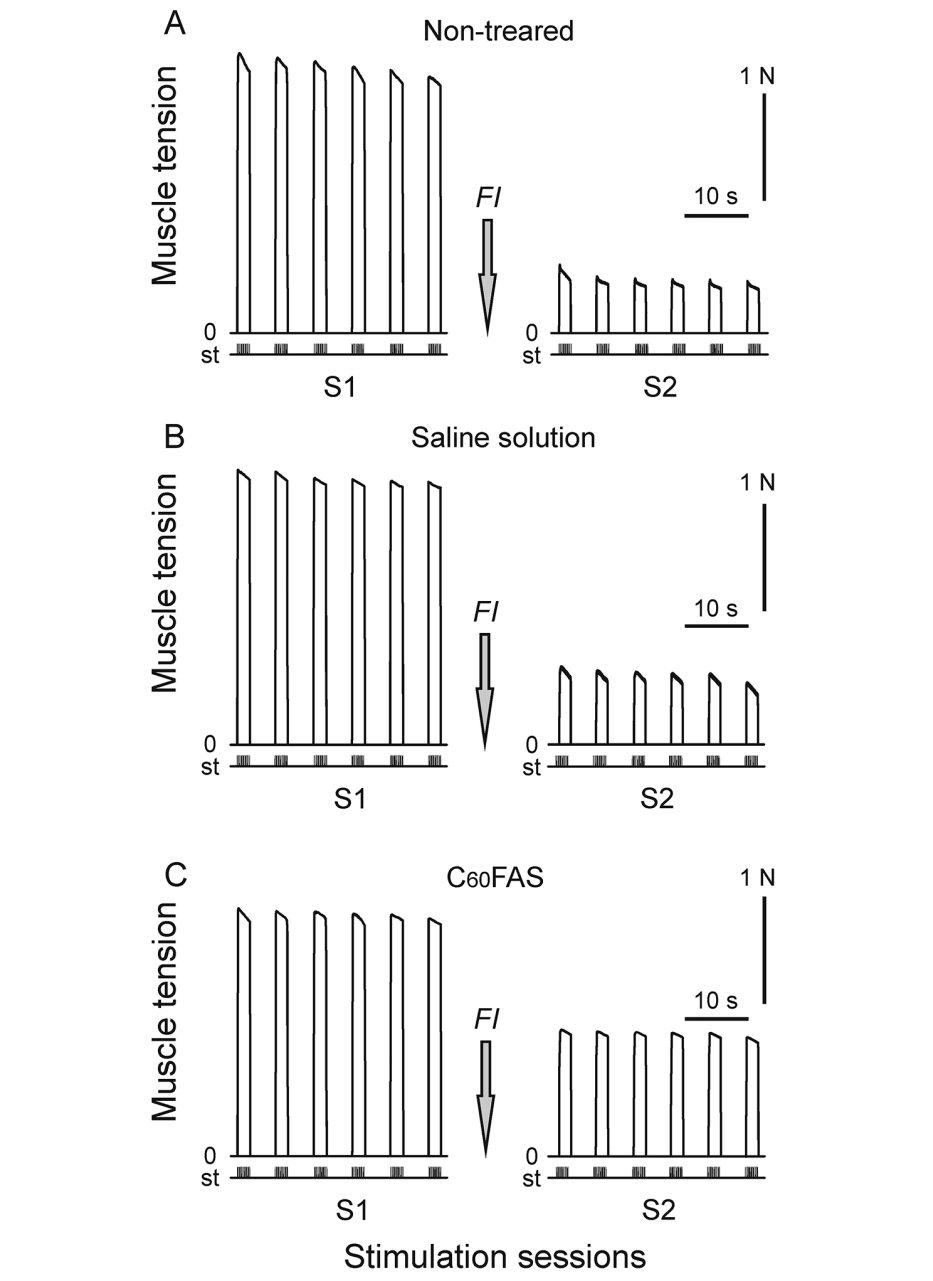
Examples of strength of the triceps surae muscle contractions during first (S1) and second (S2) session of electrical stimulations of the tibial nerve in non-treated (**A**), saline solution-treated (**B**) C_60_FAS-treated animals (**C**). FI – formalin injection; N – muscle force in Newton, st – stimulation mark

**Fig. 5:**
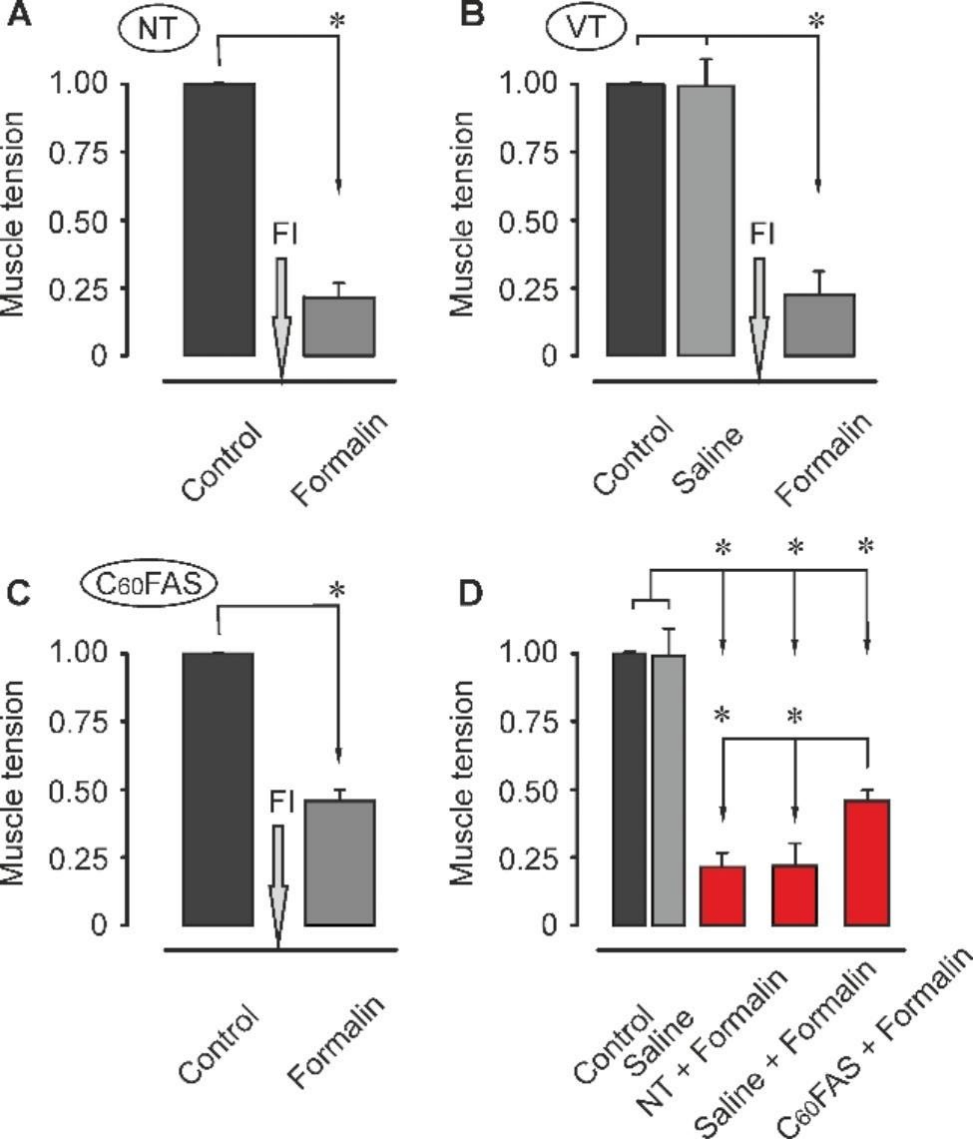
Normalized mean values ± SD of triceps surae (TS) muscle tension performed by non-treated (NT) (**A**), vehicle-treated (VT) (**B**) and fullerene-treated (C_60_FAS) (**C**) groups of rats. (**D**) Integrated data of normalized mean values of TS muscle contraction force obtained from all groups of animals. Asterisks represent significant differences (*p* < 0.05) between strength of muscle contractions before and after formalin injection (FI).

During first electrical stimulation session it was found that the level of force TS muscle contraction did not differ statically among rat of all group, *p* > 0.05, (Fig. 4A–C left column and 5A–D). However, in response to formalin administration, force of the TS muscle contraction in rats of group 3 (during second stimulation session) is decreased by an average of 77% compared with the control (Fig. 5A and B). For example, after injection of formalin, force TS muscle contraction one of the rat of group 1 was decreased from 2.6 to 0.6 N (the decrease in muscle tension was 76.9%, *p* < 005, Fig. 4A). Note that no significant difference was registered between the values of the first and second stimulation sessions in animals of group 2. It should also be noted that during second stimulation session there was not registered significant difference between values obtained from animals with injection of formalin which subjected prior saline solution administration and rats with only formalin injection (group 3 and 4, respectively), *p* > 005, (Fig. 4A,B right column and 5A,B and D). In animals of C_60_FAS-pretreated group, the dynamics of TS muscle force contraction was differ from the non-pretreated or vehicle-injected animal groups was observed. For instance, one of the rats from the group 3 after prior injection of the C_60_FAS showed decrease in TS muscle tension from 2.3 to 1.2 N (the decrease in muscle force was 46.5%, Fig. 4C). On average in the group, the decrease in the strength of muscle contraction was 48%, *p* < 0.05, (Fig. 5C and D). Thus, animals of all groups (with injection of formalin) in second stimulation session showed significant decrease in mean values of the strength TS muscle contraction in comparison with the first control session or non-treated animals. Nevertheless, the decrease in muscle contraction force under C_60_FAS application was significantly less. One-way ANOVA was used to determine the effect of C_60_FAS on muscle inflammation. So, effect of injection of formalin or injection of C_60_FAS prior application of formalin in comparison with the control was F_1,10_ = 277.73, *p* < 0.001 and F_1,10_ = 145.49, *p* < 0.001, respectively. The rats injected with saline solution not exhibited any statistically differences in changing of TS muscle tension in comparison with the formalin administration animals: F_1,10_ = 0.07, *p* = 0.8. However, statistically significant differences in force TS muscle contraction (second stimulation session) were registered between animal groups with formalin and C_60_FAS injections (F_1,10_ = 79.64, *p* < 0.001).

## Discussion

In the study, we investigate effect of C_60_FAS on TS muscle force contraction under formalin-induced inflammatory pain. In rodents, formalin pain model is described as a biphasic behavior response. Classically, it consists of the early phase that occurs during 0–10 min and a late phase that occurs during 20–60 min, as well as an interphase that occurs during 10–20 min [23, 24, 31, 32]. The duration of the phases in some authors slightly differ (± 5 min), it may be related to the dose of formalin and the injection site. However, the order of the phases remains unchanged. Pharmacological studies have determined the involvement of the inflammatory process during the second phase [31, 33]. This phase involves inflammatory mediators such as bradykinin, histamine, substance P, etc., and can be inhibited by anti-inflammatory drugs [34]. It was also noted an increase in the level of *c-fos* expression (a marker of neuronal activation) within the spinal cord, as well as activation of microglia during the second phase [31]. Thus, it should be noted that our studies took place during the second phase and correspond to the inflammatory pain model.

A prominent increase in muscle tension in animals with a preliminary C_60_FAS application under acute muscle inflammation was registered in the study. Presumably, this effect is associated with the specific antioxidant properties of C_60_ fullerene. In our previous electrophysiological and biochemical studies were shown that C_60_FAS application induces a significant decrease in lactic acid level, thiobarbituric acid reactive substances, hydrogen peroxide and ROS formation during muscle fatigue development as well as after muscle atrophy or ischemia [18–21]. C_60_ molecule is known to be a powerful scavenger of ROS due to its ability to bind electrons [35], and excessive accumulation of ROS (oxidative stress) is closely related to the process of acute and chronic inflammation [36]. In this regard, it is interesting that various derivatives of C_60_ fullerene and especially water-soluble C_60_ fullerenes have a protective effect, for example, protect cell growth from various toxins, and also can promote recovery after ischemic stroke [37–39]. Pei et al. [13] have shown that water-soluble polyhydroxylated C_60_ fullerene can inhibit the inflammatory response by reducing ROS production and down-regulating expression of inflammatory genes possibly via ROS/p38 MAPK/NFkB and ROS/p38 MAPK/FoxO1 pathways, and can inhibit progression of experimental osteoarthritis. It was also demonstrated that water-soluble C_60_ fullerenes decrease in the level of c-Fos immunoreactivity within laterocapsular division of the central nucleus of amygdala (also known as the “nociceptive amygdala”) under skeletal muscle fatigue development [40]. Yamada et al. [41] in turn showed that water-soluble C_60_ fullerene does not penetrate the blood-brain barrier. Thus, we can say that the area of influence of C_60_FAS is limited from central and peripheral only to peripheral and its anti-inflammatory properties are obviously associated with a decrease in ROS production in tissue.

## Conclusion

Based on the results of this investigation and results of our previous electrophysiological, and biochemical studies (described above) the following assumptions can be made. We suggest that in conditions of acute inflammatory pain, an increase in the strength of muscle contractions (which occurred in the case of C_60_FAS application) is associated with a decrease in the concentration of free radicals in the inflamed muscle tissue and indicates the activation of the protective effect of the antioxidant system in response to formalin administration. In this regard, it can be speculated that water-soluble C_60_ fullerene may potentially be used as an anti-pain and anti-inflammatory agent, which, by reducing the amount of free radicals in the muscle, reduces the transmission intensity of nociceptive information from the inflamed muscle to the spinal cord and the brain, and thereby improves the functional state of skeletal muscle.

## Data Availability

The datasets generated during the current study are available from the corresponding author on reasonable request.

## Abbreviations

ADC: Analogue to digital converter
ANOVA: Analysis of variance
AFM: Atomic force microscopy
CNS: Central nervous system
C_60_FAS: C_60_ fullerene aqueous solution
DAC: Digital to analogue converter
TS: Triceps surae muscle
ROS: Reactive oxygen species
SD: Standard deviation
